# Prevalence and determinants of modern contraceptive utilization among rural lactating mothers: findings from the primary health care project in two northwest Ethiopian districts

**DOI:** 10.1186/s12905-020-00933-7

**Published:** 2020-04-03

**Authors:** Terefe Derso, Gashaw Andargie Biks, Mezgebu Yitayal, Tadesse Awoke Ayele, Kassahun Alemu, Getu Debalkie Demissie, Haileab Fekadu Wolde, Endalkachew Dellie, Telake Azale, Bisrat Misganaw, Adane Kebede, Destaw Fetene, Tsegaye Gebremdhin, Asmamaw Atnafu

**Affiliations:** 1 Department of Human Nutrition, Institute of Public Health, College of Medicine and Health Sciences, University of Gondar, Gondar, Ethiopia; 2 Dabat Research Centre Health and Demographic Surveillance System, Institute of Public Health College of Medicine and Health Science, University of Gondar, Gondar, Ethiopia; 3 Department of Health Systems and Policy, Institute of Public Health, College of Medicine and Health Sciences, University of Gondar, Gondar, Ethiopia; 4 Department of Epidemiology and Biostatics, Institute of Public Health, College of Medicine and Health Sciences, University of Gondar, Gondar, Ethiopia; 5 Department of Health education and behavioral sciences, Institute of Public Health, College of Medicine and Health Sciences, University of Gondar, Gondar, Ethiopia

**Keywords:** Contraceptive utilization, Postnatal care, Child immunization

## Abstract

**Background:**

Contraceptive utilization is a guarantee to avert unwanted pregnancies. In Ethiopia however, more than half of the rural women have shorter birth intervals. Consequently, 17 and 8% of the births have been either mistimed (wanted at later date) or unwanted, respectively. Therefore, this study investigated modern contraceptive utilization and its predictors among rural lactating women.

**Methods:**

A community based-cross-sectional study was conducted from May 01 to June 29, 2019, in Dabat and Gondar zuria districts, northwest Ethiopia. Data from 603 lactating mother were collected through face to face interviews using a structured questionnaire. Bivariate and multivariate logistic regression analyses were fitted to identify the independent predictors of modern contraceptive utilization.

**Results:**

The overall prevalence of modern contraceptive (MC) utilization rate was 45.8% [95% CI: 38.01, 53.59]. The contraceptive method mix was dominated by Depo-Provera (39.8%) followed by implants (4.8%). The odds of utilization of contraceptive were 5.58 times higher among mothers of children with fully immunized [AOR = 5.58, 95% CI: 3.45, 9.01] compared to mothers whose children were vaccinated partially or not at all. Mothers who received antenatal [AOR = 1.74, 95% CI: 1.13, 4.43] and postnatal care [AOR = 2.02, 95%CI: 1.24, 2.91) were 1.74 and 2.02 folds more likely to utilize modern contraceptives than mothers who did not receive such care, respectively.

**Conclusion:**

The prevalence of modern contraceptive utilization in this study area was lower than the planed national target. In the region, child immunization service is one of the promising platforms for reaching lactating mothers with modern contraceptive utilization. Our findings suggest that antenatal and postnatal care visits are the other key determinants of modern contraceptive utilization. Thus, in low-resource settings like ours, the health system approaches to improved antenatal and, postnatal care and child immunization services should be intensified with more effective advice on modern contraceptive utilization to reduce unwanted pregnancies.

## Background

The health benefits of contraceptive use include preventing unplanned pregnancies [1] and averting maternal and child morbidity by allowing couples to space their pregnancies by more than two years [2–4]. In spite of this, more than half of the lactating women are at high risk for unwanted or unplanned pregnancy immediately after birth [5]. The postpartum period, the time of care for themselves and their newborns provides an important opportunity for contacting the health system [6]. In fact, postpartum modern contraceptive has the potential of reduce 71% of unwanted and abolishing 53 million of the unintended pregnancies as well as resulting in 22, 25 and 7 million fewer unplanned births, induced abortion and miscarriages, respectively [7]. Also, longer birth intervals or contraceptive uses can avert about 1 in 5 deaths in children of 1 to 4 years of age [8] and 44.3% of maternal deaths [9].

Since 1993 Ethiopia has been implementing a clear population policy [10], and one of the major strategies of the policy currently the family planning program to raise contraceptive prevalence to 55% by 2020 through free of charge provisions at both government and non-government health facilities [11, 12]. So far, modern contraceptive prevalence is low (35%) with significant regional variations, like for example, Somali (1%), Affar (12%) and Amhara [47%]. Consequently, 17 and 8% of the births were mistimed (wanted at later date) and unwanted, respectively [13].

Contraceptive uptake has been positively associated with a range of both health and non-health related outcomes. Factors that influence contraceptive practice are multifaceted and complex. Previous studies shown that socio-cultural, health and socio-economic characteristics are main significant determinants of modern contraceptive utilization. High contraceptive utilization has been documented among literate [13, 14], old age [14], rural residences [13], media familiar and richest women [15] who have had many (> 5) live children [16, 17]. Health care utilizations during pregnancy and after delivery, antenatal and postnatal care [18, 19] and child immunization [20] also increase the likelihood of modern contraceptive utilization.

Mothers wish to space their births sufficiently far apart that even when their children a toddlers, they prefer to delay pregnancies [21]. However, in Ethiopia more than half of the rural women have shorter birth intervals; consequently, high maternal mortality rate is documented [13]. Also subsequent conceptions and births negatively affect mothers’ decisions to continue breast-feeding children [22]. Some local studies have focused on assessing the contraceptive utilization status of reproductive women generally [16, 17], only two local studies in Ethiopia targeted to urban lactating women [23, 24]. Thus, the lactating period is a window of opportunity to improve contraceptive utilization [7]. Investigating the prevalence and predictors of modern contraceptive utilization in rural area is of a vital importance to design strategies to address the problem. Therefore, this study investigated modern contraceptive utilization and its predictors among rural lactating women.

## Methods

### Study setting and design

A community-based cross-sectional study was carried out from May 01 to June 29, 2019 in Dabat and Gondar Zuria districts, northwest Ethiopia. Two of the total 23 districts in North Gondar zone of the Amhara region, Dabat and Gondar zuria districts, consist of 30 and 38 kebeles (the smallest administrative units in Ethiopia), respectively. Located in different ecological zones (high, middle, and low land), the districts had 145,509(Dabat) and 231,324(Gondar zuria) inhabitants who’s largely depended on subsistence farming. Of the total inhabitants, 5973 in Dabat and 8180 in Gondar zuria district were lactating mothers, respectively.

### Study population and sampling procedure

Lactating mothers who lived in Dabat and Gondar zuria districts for at least 6 months were included. The study aimed to assess the prevalence and determinants of modern contraceptive utilization among rural lactating mothers in Dabat and Gondar zuria districts, northwest Ethiopia. Of the total kebeles, eight in Dabat and 10 in Gondar zuria were selected using the lottery method. The systematic sampling technique was used to select study participants. For households with multiple lactating mothers who fulfilled the inclusion criteria, the lottery method was used to choose one.

Sample size was calculated using Epi-info version 3.7 by considering the assumptions: 48.4% prevalence of modern contraceptive utilization in Gondar town [23, 25], 95% level of confidence and 5% margin of error. A design effect of 1.5 and 10% non-response rate were also anticipated to obtain the final sample size of 631.

### Data collection tools and procedure

A structured questionnaire was adapted from the Ethiopian Demography and Health Survey (EDHS) to collect the data [8, 13]. The questionnaire was composed of socio-demographic characteristics and primary health care utilization (immunization, family planning, health education, hygiene and sanitation, maternal health service and physical access to health services) [13, 25]. The questionnaire was first prepared in English and translated to Amharic (the native language of the study area). Back translation to English was made to compare the consistency and amendments accordingly. Fifteen data collectors and three field supervisors were recruited for data collection. Two days training was given on the objective of the study, confidentiality of information and the techniques of an interview to data collectors and supervisors.

### Study variables and data analysis

To ascertain the outcome variable, modern utilization of contraceptive, women were asked if they were currently using a method of contraception and what method they were using. The outcome was coded 1 if they were using a modern method (pill, Intra uterine contraceptive device (IUCD), injections, condom, male or female sterilization, implant, or diaphragm/foam/jelly) and 0 if they were using a traditional method or were not currently using a method. Epi-data version 3.1 was used for data entry and data were exported to SPSS version 21 for analysis. Descriptive statistics were computed. Binary Logistic regression model was used to identify the relationship between dependent and independent variables. Those significant independent variables in bivariate analysis (*p*-value< 0.2) were entered into the multivariable analysis. In the binary logistic regression model, backward- stepwise multivariate analysis was used to elicit associated factors of modern utilization of contraceptive. In the final model, a significant association was declared at a *p*-value less than 0.05 and finally, the results were presented in texts and tables with adjusted odds ratio (AOR) and the corresponding 95% confidence interval.

## Results

### Socio-demographic characteristics of study participants

A total of 603 mothers (with a response rate of 95.56%) participated in the study. Most of mothers (94.7%) were housewives. Only nearly one-fourth (22.1%) had formal education; 22.9% were in the age ranges of 36–49 years (Table 1).

**Table 1:** Socio-demographic characteristics of study participants in the rural population of northwest Ethiopia

Characteristics	Frequency	Percent
Age of mothers (in years)		
18–24	102	16.9
25–35	363	60.2
36–49	138	22.9
Maternal educational status		
Unable to read and write	400	66.3
Able to read and write without formal education	70	11.6
Formal education	133	22.1
Husband’s educational status		
Unable to read and write	344	57.0
Able to read and write without formal education	133	22.1
First cycle(1–4 grade)	41	6.8
Second cycle(5–8 grade)	50	8.3
Secondary school(9–12 grade)	26	4.3
Certificate and above	9	1.5
Occupation of mothers		
House wife	571	94.7
Outdoor worker	32	5.3
Number of children		
1–4	422	70.0
Above 4 children	181	30.0

### Health related characteristics of study participants

Of all participants, 95.2% had antenatal care (ANC) visits; 70.0% delivered in health institutions (Table 2).

**Table 2:** Health related characteristics of study participants in the rural population of northwest, Ethiopia

Characteristics	Frequency	Percent
Travel time to the nearest health center (HC)		
30 or below minutes	146	24.2
Above 30 min	457	75.8
ANC visit during your last pregnancy		
Yes	574	95.2
No	29	4.8
Place of last delivery		
Home	181	30.0
Health institution	422	70.0
Postnatal care in the current delivery		
Yes	397	65.8
No	206	34.2
Place of postnatal care		
Hospital	22	3.6
Health Center	267	44.3
Health Post	108	17.9
Child immunization status		
Fully vaccinated	464	76.9
Partially/not vaccinated	139	23.1

### Prevalence of modern contraceptive utilization

The prevalence of modern contraceptive (MC) utilization was 45.8% [95% CI: 38.01, 53.59]. The contraceptive method mix was dominated by Depo-Provera (39.8%) followed by implants (4.8%). However, very few mothers (1.2 and 0.2%) utilized pills and Intra uterine contraceptive devices, respectively.

### Factors associated with modern contraceptive utilization

In the bivariable analysis age of mothers, number of children, place of last delivery, travel time to the nearest health center, antenatal care visit, postnatal care and child immunization status were found associated with a p-value of less than 0.2. However, the result of the multivariable analysis revealed with a *p*-value of less than 0.05 that the odds of modern utilization of contraceptive were 5.58 times higher among with children with fully immunization [AOR = 5.58, 95% CI: 3.45, 9.01] compared to mothers with children with incomplete or not vaccinated. Mothers who received antenatal care [AOR = 1.74, 95% CI: 1.13, 4.43] and postnatal care [AOR = 2.02, 95%CI: 1.24, 2.91) were 1.74 and 2.02 folds more likely to utilize modern contraceptive than mothers who did not received antenatal care and postnatal care, respectively (Table 3).

**Table 3:** Factors associated with modern utilization of contraceptive in the rural population of northwest, Ethiopia

Variables	Modern utilization of contraceptive		Crude Odds Ratio (95%CI)	Adjusted Odds Ratio (95%CI)
	Yes	No		
Age of mothers (in years)				
18–24	39	63	1	
25–35	171	192	1.44 (0.92, 2.26)	
36–49	66	72	1.48 (0.88, 2.49)	
Place of last delivery				
Home	70	111	1	1
Health institution	206	216	1.51 (1.06, 2.16)	
Antenatal care visit				
Yes	269	305	2.77 (1.17, 6.59)	1.74 (1.13, 4.43)*
No	7	22	1	1
Postnatal care				
Yes	206	191	2.09 (1.48, 2.97)	2.02 (1.24, 2.91)*
no	70	136	1	1
Child immunization status				
Fully vaccinated	252	212	5.69 (3.54, 9.17)	5.58 (3.45, 9.01)*
Partially/not vaccinated	24	115	1	1
Number of children			1	
1–4	191	231		
> 4	85	91	1.07 (0.76,1.52)	

## Discussion

Contraceptives utilization is a guarantee to avert unwanted pregnancies [1]. This community based cross-sectional study revealed that the overall prevalence of modern contraceptive utilization was 45.8% [95% CI: 38.01, 53.59]; antenatal and postnatal care; and child immunization status were the predictors of the utilization of contraceptives. In this study the magnitude of modern contraceptive utilization was consistent with that of a previous study reported from Gondar city (48.4%) [23].

However, the magnitude of modern contraceptive utilization in our study is lower than the finding in Malawi where three-fourths (75%) of the lactating women utilized contraceptives [26]. This difference might be due to variations in the awareness of the people, the availability of the contraceptive methods and differences in study settings to access the service. The high prevalence in Malawi compared to ours might be due to differences the education levels. In our study more than three-fourths (77.9%) of the participants were illiterate, while in Malawi, the great majority (90.2%) of the participants had primary and above education. Since education enables mothers to have knowledge and awareness about the benefits of family planning, it enhances their modern contraceptive utilization. This implies that educated women have greater ability to stick to health care inputs, such as modern contraceptive utilization which improves birth spacing.

Mothers who received antenatal care were 1.74 folds more likely to utilize modern contraceptive than mothers who did not received such care. These higher odds of modern contraceptive utilization are supported by findings reported from Ethiopia [19] and Nigeria [18]. The plausible reason might be that during antenatal care follow ups the mothers obtain counseling on birth spacing and family planning. Thus, antenatal care is important to reach women with information about the return of fertility, their options to space or limit future pregnancies, and the benefits to their own and their newborn’s health of doing so before they are at risk for unintended pregnancies. Recommending that women be counseled on birth spacing and family planning during ANC contacts is worth while.

In the multivariate analysis, postnatal care was the other significant variable because mothers who received postnatal care were 2.02 times more likely to utilize modern contraceptive than their counterparts as repeated by other studies in Africa [18, 19]. This might be so because postnatal care enabled health workers to counsel women on the importance of birth spacing. Therefore, postnatal care should be prioritized with more effective advice on family planning to reduce unintended births.

The uncommon finding of this study showed that the likelihood of modern contraceptive utilization was 5.58 times higher among lactating mothers with fully immunized children compared to mothers of who were either partially immunized or not vaccinated at all. In fact, the recommended vaccination schedule for children allows for multiple health care contacts with infants and their mothers during the first year of life [20]. These routine immunization services offer important opportunities to discuss family planning and to reach many postpartum women during group education talks. In Ethiopia, immunization contacts occur regularly according to the national schedule of 6, 10, 14 weeks and 9 and 15 months [27]. Thus, child immunization services are promising Platforms to reach lactating mothers with modern contraceptive utilization [28]. Finally, advocacy efforts can promote positive partnerships between child health and family planning programs as a ‘win-win’ for the health of both the mother and the infant.

Limitation: This study provides important insights about modern contraceptive utilization and its predictors among lactating mothers in Dabat and Gondar Zuria districts, where there is a scarcity of literature. However, the cross sectional nature of the study has limited its capacity of measuring the cause and effect relationships of the outcome and potential predictors.

Conclusion: In the study area, the prevalence of modern contraceptive utilization was lower documented compared to the planed national figure. In the region, child immunization services were promising platforms to reach lactating mothers with modern contraceptives. Also our findings suggest that antenatal and postnatal care visits are the other key determinants of modern contraceptive utilization. In low-resource settings like Ethiopia, the health system approaches to improved antenatal, postnatal care and child immunization services should be intensified with more effective advice on modern contraceptive utilization to reduce unwanted pregnancies.

## Data Availability

Data will be made available upon request to the primary/ corresponding author.

## Abbreviations

AOR: Adjusted Odds Ratio
ANC: Antenatal Care
COR: Crude Odds Ratio
CI: Confidence Interval
IUCD: Intra Uterine Contraceptive Device
PNC: Postnatal Care
SPSS: Statistical Package for Social Science
WHO: World Health Organization
